# Potent Anti‐SARS‐CoV‐2 Efficacy of COVID‐19 Hyperimmune Globulin from Vaccine‐Immunized Plasma

**DOI:** 10.1002/advs.202104333

**Published:** 2022-04-11

**Authors:** Ding Yu, Yu‐Feng Li, Hong Liang, Jun‐Zheng Wu, Yong Hu, Yan Peng, Tao‐Jing Li, Ji‐Feng Hou, Wei‐Jin Huang, Li‐Dong Guan, Ren Han, Yan‐Tao Xing, Yong Zhang, Jia Liu, Lu Feng, Chun‐Yan Li, Xiao‐Long Liang, Ya‐Ling Ding, Zhi‐Jun Zhou, De‐Ming Ji, Fei‐Fei Wang, Jian‐Hong Yu, Kun Deng, Dong‐Mei Xia, De‐Mei Dong, Heng‐Rui Hu, Ya‐Jie Liu, Dao‐Xing Fu, Yan‐Lin He, Dong‐Bo Zhou, Hui‐Chuan Yang, Rui Jia, Chang‐Wen Ke, Tao Du, Yong Xie, Rong Zhou, Ce‐Sheng Li, Man‐Li Wang, Xiao‐Ming Yang

**Affiliations:** ^1^ Chengdu Rongsheng Pharmaceuticals Co. Ltd. Chengdu 610041 China; ^2^ Beijing Tiantan Biological Products Co. Ltd. Beijing 100024 China; ^3^ Center for Biosafety Mega‐Science Wuhan Institute of Virology Chinese Academy of Sciences Wuhan 430071 China; ^4^ Sinopharm Wuhan Plasma‐derived Biotherapies Co. Ltd. Wuhan 430207 China; ^5^ National Institute for Food and Drug Control of China Beijing 102629 China; ^6^ China National Biotec Group Company Limited Beijing 100029 China; ^7^ Guangdong Provincial Center for Disease Control and Prevention Guangzhou 511430 China

**Keywords:** COVID‐19 hyperimmune globulin, passive immunotherapy, SARS‐CoV‐2 variant, sinopharm COVID‐19 vaccine

## Abstract

Coronavirus disease 2019 (COVID‐19) remains a global public health threat. Hence, more effective and specific antivirals are urgently needed. Here, COVID‐19 hyperimmune globulin (COVID‐HIG), a passive immunotherapy, is prepared from the plasma of healthy donors vaccinated with BBIBP‐CorV (Sinopharm COVID‐19 vaccine). COVID‐HIG shows high‐affinity binding to severe acute respiratory syndrome coronavirus 2 (SARS‐CoV‐2) spike (S) protein, the receptor‐binding domain (RBD), the *N*‐terminal domain of the S protein, and the nucleocapsid protein; and blocks RBD binding to human angiotensin‐converting enzyme 2 (hACE2). Pseudotyped and authentic virus‐based assays show that COVID‐HIG displays broad‐spectrum neutralization effects on a wide variety of SARS‐CoV‐2 variants, including D614G, Alpha (B.1.1.7), Beta (B.1.351), Gamma (P.1), Kappa (B.1.617.1), Delta (B.1.617.2), and Omicron (B.1.1.529) in vitro. However, a significant reduction in the neutralization titer is detected against Beta, Delta, and Omicron variants. Additionally, assessments of the prophylactic and treatment efficacy of COVID‐HIG in an Adv5‐hACE2‐transduced IFNAR^−/−^ mouse model of SARS‐CoV‐2 infection show significantly reduced weight loss, lung viral loads, and lung pathological injury. Moreover, COVID‐HIG exhibits neutralization potency similar to that of anti‐SARS‐CoV‐2 hyperimmune globulin from pooled convalescent plasma. Overall, the results demonstrate the potential of COVID‐HIG against SARS‐CoV‐2 infection and provide reference for subsequent clinical trials.

## Graphical abstract

Coronavirus disease 2019 (COVID‐19) hyperimmune globulin (COVID‐HIG) prepared from the plasma of healthy donors vaccinated with BBIBP‐CorV (Sinopharm COVID‐19 vaccine) exhibits broad‐spectrum neutralization effects against multiple severe acute respiratory syndrome coronavirus 2 (SARS‐CoV‐2) variants of concern in vitro and shows protective effect against SARS‐CoV‐2 infection in vivo. It is currently under clinical evaluation for the treatment of COVID‐19 (NCT05173441).

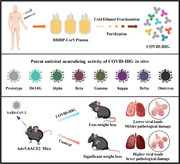

## 1 Introduction

Coronavirus disease 2019 (COVID‐19) is caused by the severe acute respiratory syndrome coronavirus 2 (SARS‐CoV‐2). The estimated COVID‐19 basic reproduction number (*R*_0_) varies between 2.2 and 3.9.^[^ ^1^ ^]^ Owing to the widespread infection, genetic variants of the virus have appeared in an increasing number of countries, including the Alpha (501Y.V1, B.1.1.7),^[^ ^2^ ^]^ Beta (501Y.V2, B.1.351),^[^ ^3^ ^]^ Gamma (501Y.V3, P.1),^[^ ^4^, ^5^ ^]^ Kappa (B.1.617.1), Delta (B.1.617.2),^[^ ^6^ ^]^ and Omicron (B.1.1.529) variants.^[^ ^7^ ^]^ These variants make epidemic prevention and control even more challenging.

Amino acid changes in viral surface proteins can greatly alter viral function and/or interactions with antibodies.^[^ ^8^ ^]^ The SARS‐CoV‐2 spike (S) protein binds to human angiotensin‐converting enzyme 2 (hACE2) through its receptor‐binding domain (RBD) to allow SARS‐CoV‐2 to efficiently enter cells.^[^ ^9^ ^]^ The Beta variant is characterized by eight lineage‐defining mutations in the S protein that may have functional relevance.^[^ ^10^ ^]^ Notably, the results of the clinical trials of three COVID‐19 vaccines (Novavax NVX‐CoV2373, Janssen Ad26.COV2.S, and AstraZeneca ChAdOx1) performed in South Africa during the second wave revealed a significantly lower vaccine efficacy for the Beta variant.^[^ ^11^ ^]^

BBIBP‐CorV, an inactivated SARS‐CoV‐2 vaccine developed in China,^[^ ^12^, ^13^ ^]^ was approved for emergency use authorization by the World Health Organization on May 7, 2021 (https://www.who.int/news/item/07–05–2021‐who‐lists‐additional‐covid‐19‐vaccine‐for‐emergency‐use‐and‐issues‐interim‐policy‐recommendations). The antibodies induced by BBIBP‐CorV can neutralize multiple SARS‐CoV‐2 strains (including the Alpha and Beta variants), suggesting the potential of BBIBP‐CorV to provide cross‐protection against SARS‐CoV‐2 variants.^[^ ^14^, ^15^ ^]^ Shortly after the outbreak of the Beta variant, one study showed that Beta did not escape immunity induced by BBIBP‐CorV, with the geometric mean titers of serum samples from recipients of BBIBP‐CorV decreasing from 111 to 72 compared with the titers against the SARS‐CoV‐2 HB02 strain (BBIBP‐CorV development was based on the HB02 strain).^[^ ^14^ ^]^

Passive immunization is a promising strategy for preventing and controlling infectious diseases,^[^ ^16^ ^]^ and the US Food and Drug Administration approved the use of convalescent plasma (CP, a strategy of passive immunization) therapy for treating hospitalized patients with COVID‐19.^[^ ^17^ ^]^ Early studies reported significant clinical symptom improvement in patients with severe COVID‐19 after CP treatment.^[^ ^18^, ^19^ ^]^ Hospitalized patients with COVID‐19 showed a lower risk of death after transfusion of plasma with higher anti‐SARS‐CoV‐2 immunoglobulin G (IgG) antibody levels than after transfusion of plasma with lower antibody levels.^[^ ^20^ ^]^ However, a recent study on the efficacy of CP therapy in patients admitted to the hospital with COVID‐19 in the UK showed that high‐titer CP did not improve survival or other prespecified clinical outcomes.^[^ ^21^ ^]^ Thus, studies of CP therapy have shown inconsistent outcomes in the context of clinical treatment of COVID‐19.

Anti‐SARS‐CoV‐2 hyperimmune globulin (HIG), another passive immunotherapy, shows obvious advantages over CP in terms of its purity, low risk from blood‐borne viruses, unnecessary blood cross‐matching, and ease of storage and transportation. In this study, we produced COVID‐19 hyperimmune globulin (COVID‐HIG) from the plasma of humans vaccinated with BBIBP‐CorV using a commercialized method and verified the anti‐SARS‐CoV‐2 activity of COVID‐HIG. Further nonclinical pharmacodynamic studies are needed to verify the antiviral activity of COVID‐HIG prior to conducting clinical research.

## 2 Results

### 2.1 Production of COVID‐HIG from Human Plasma after BBIBP‐CorV Vaccination

**Figure 1:**
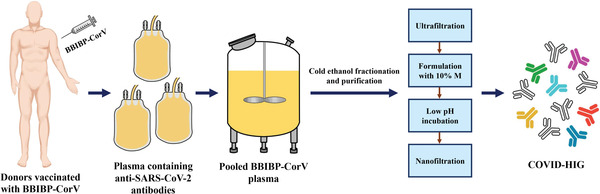
Workflow for COVID‐HIG production. Plasma containing anti‐SARS‐CoV‐2 antibodies was collected from immunized healthy donors who were vaccinated with BBIBP‐CorV (two‐dose). The plasma samples were combined to obtain pooled BBIBP‐CorV plasma. Then, pooled BBIBP‐CorV plasma was fractionated and purified to prepare COVID‐HIG by commercial cold ethanol fractionation. M, maltose. COVID‐HIG, COVID‐19 hyperimmune globulin.

We first collected plasma from healthy donors who had been immunized with two doses of BBIBP‐CorV. The plasma samples were combined to obtain pooled BBIBP‐CorV plasma (PBP). The PBP was fractionated and purified to prepare COVID‐HIG (**Figure** **1**). We prepared three batches of COVID‐HIG with final batch numbers of COVID‐HIG‐001 (34 combined plasma samples), COVID‐HIG‐002 (840 combined plasma samples), and COVID‐HIG‐003 (1502 combined plasma samples).

The composition of PBP is variable and complex. In addition to the anti‐SARS‐CoV‐2 antibodies, PBP contains inorganic salts, organic compounds, water, and various proteins. Common plasma contains more than 1000 protein types, such as complement proteins, coagulation factors, and antithrombotic factors.^[^ ^22^ ^]^ Furthermore, unlike PBP, COVID‐HIG does not contain inorganic compounds, and the IgG component showed >96% purity (Table S1, Supporting Information). The average IgG concentration (three batches of COVID‐HIG) was increased by 5.5‐fold compared to that of PBP (Table S1, Supporting Information). The detected IgG subtypes and their proportions were IgG1 (65.9%), IgG2 (28.9%), IgG3 (3.1%), and IgG4 (2.1%) (Table S2, Supporting Information).

### 2.2 COVID‐HIG Binds to SARS‐CoV‐2 S Protein, the *N*‐Terminal Domain (NTD) of S protein, Nucleocapsid Protein (NP), and RBD; Competes with hACE2 for RBD Binding

**Figure 2:**
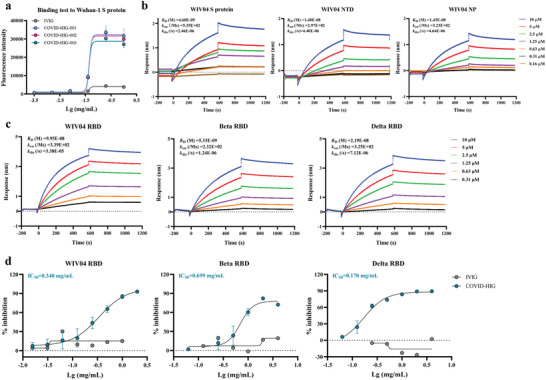
In vitro affinity and competition assays of COVID‐HIG. a) Fluorescence intensity of different concentrations of COVID‐HIG (COVID‐HIG‐001, ‐002, and ‐003) and IVIG respond to CHO‐K1/Wuhan‐1 S cells. IVIG was used as a control. Values are presented as the mean ± standard deviation (SD) of three technical replicates (*n* = 3). b) COVID‐HIG showed high‐affinity binding to the SARS‐CoV‐2 WIV04 S protein, the NTD, and NP; and c) WIV04, Beta, and Delta RBDs in biolayer interferometry (BLI) assays. We performed BLI experiments for twice and one representative result data is shown. d) Competitive enzyme‐linked immunosorbent assays (ELISAs) were conducted to determine the blocking potency of COVID‐HIG in the RBD (WIV04, Beta, and Delta)‐hACE2 interactions. IVIG was used as a control. Values are presented as the mean ± standard error (SEM) of three technical replicates (*n* = 3). Wuhan‐1, SARS‐CoV‐2 Wuhan‐Hu‐1 strain. WIV04, nCoV‐2019BetaCoV/Wuhan/WIV04/2019 strain. NTD, *N*‐terminal domain of S protein. NP, nucleocapsid protein. RBD, receptor‐binding domain of S protein. *K*_D_, equilibrium dissociation constant. *k*_on_, association rate constant. *k*_dis_, dissociation rate constant. To be noted, since COVID‐HIG consists of multiple antibodies, the *K*_D_ value does not represent a real affinity. The average affinity of the minority of specific antibodies with many epitope specificities remains unknown. hACE2, human angiotensin‐converting enzyme 2. IVIG, human immune globulin intravenous. IC_50_, 50% inhibitory concentration.

The S protein on the SARS‐CoV‐2 surface allows the virus to enter cells by binding to the ACE2 receptor on susceptible cells.^[^ ^23^ ^]^ Therefore, we first determined whether COVID‐HIG could effectively bind to the S protein. The results of fluorescence‐activated cell sorting (FACS) revealed that COVID‐HIG effectively bound to CHO‐K1 cells stably expressing the SARS‐CoV‐2 Wuhan‐Hu‐1 (abbreviated as Wuhan‐1) strain^[^ ^24^ ^]^ S protein, with an average half‐maximal effective concentration value of 0.052 mg mL^−1^ (COVID‐HIG‐001: 0.053, COVID‐HIG‐002: 0.053, COVID‐HIG‐003: 0.051 mg mL^−1^) (**Figure** **2**a). To determine the binding affinity of COVID‐HIG with the S protein, the NTD, NP, and RBD of SARS‐CoV‐2, biolayer interferometry (BLI) experiments were conducted. COVID‐HIG showed high‐affinity binding to the full‐length S protein, the NTD, and NP of the SARS‐CoV‐2 nCoV‐2019BetaCoV/Wuhan/WIV04/2019 (WIV04) strain;^[^ ^25^ ^]^ the measured equilibrium dissociation constant (*K*_D_) values were 4.60 × 10^−9^, 14.8 × 10^−9^, and 14.3 × 10^−9^ m, respectively (Figure 2b and Table S3, Supporting Information). In addition, COVID‐HIG displayed specific and high affinity to SARS‐CoV‐2 WIV04, Beta, and Delta RBDs, with *K*_D_ values both lower than 100 × 10^−9^ m (Figure 2c and Table S3, Supporting Information). Next, we determined whether COVID‐HIG could prevent SARS‐CoV‐2 RBD binding to hACE2. Competitive enzyme‐linked immunosorbent assay (ELISA) assays were performed to calculate the average 50% inhibitory concentration (IC_50_) values for the blocking ability of COVID‐HIG. COVID‐HIG effectively blocked the binding between hACE2 and SARS‐CoV‐2 WIV04, Beta and Delta RBDs. In the presence of COVID‐HIG, nearly 90% inhibition was achieved, suggesting that COVID‐HIG contained anti‐SARS‐CoV‐2 antibodies targeting the RBD‐binding site of SARS‐CoV‐2 WIV04 and variants (Figure 2d). As a negative control, human immune globulin intravenous (IVIG) showed no blocking activity (Figure 2d).

### 2.3 COVID‐HIG Neutralizes a Wide Variety of Spike SARS‐CoV‐2 Pseudotyped Viruses

For highly pathogenic viruses, pseudotyped viruses have become useful virological tools due to their safety and versatile properties. To verify whether SARS‐CoV‐2 strains with key mutations in the S protein region can escape COVID‐HIG, different vesicular stomatitis virus‐based SARS‐CoV‐2 pseudotyped viruses were used to determine the neutralizing titer of COVID‐HIG using pseudotyped virus‐based neutralization assays (PBNAs).^[^ ^26^ ^]^

**Figure 3:**
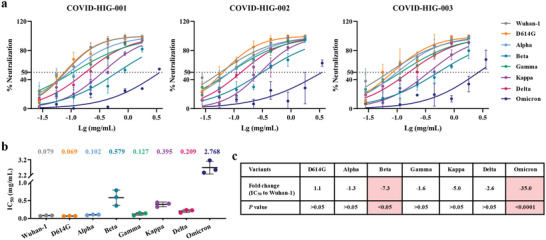
Antiviral activity of COVID‐HIG against SARS‐CoV‐2 pseudotyped viruses in vitro. a) All three batches of COVID‐HIG have potent neutralization potency against the eight pseudotyped SARS‐CoV‐2 virus strains. Pseudotyped viruses were preincubated with serial dilutions of COVID‐HIG at different concentrations for 1 h at 37 °C. Next, Huh‐7 cells were incubated with the pseudotyped viruses for 24 h. Luciferase was detected to assess infection. The *y*‐axis represents percent inhibition. Data are shown as mean ± SD of three independent experiments (*n* = 3). b) Comparison of the IC_50_ for the pseudotyped SARS‐CoV‐2 variants from the pseudotyped Wuhan‐1 strain. The IC_50_ (mg mL^−1^) of COVID‐HIG against Wuhan‐1 or seven spike variants of SARS‐CoV‐2 is shown and marked on top of each group and lined with SD shown as error bars. c) Summary of the fold‐change in neutralization potency and *P*‐value of the IC_50_ for the pseudotyped SARS‐CoV‐2 variants in relation to the pseudotyped Wuhan‐1 strain. The light red background indicates significantly decreased neutralization potency of COVID‐HIG against pseudotyped SARS‐CoV‐2 variants compared with that of the pseudotyped Wuhan‐1 strain. Statistical significance was determined using one‐way ANOVA.

The pseudotyped viruses included the Wuhan‐1, D614G mutant, Alpha, Beta, Gamma, Kappa, Delta, and Omicron variants. The results revealed that all three batches of COVID‐HIG (COVID‐HIG‐001, ‐002, and ‐003) had potent neutralizing activities (**Figure** **3**a). The average 50% inhibitory concentration (IC_50_) values of three batches of COVID‐HIG against these eight pseudotyped SARS‐CoV‐2 virus strains were 0.079 (Wuhan‐1), 0.069 (D614G), 0.102 (Alpha), 0.579 (Beta), 0.127 (Gamma), 0.395 (Kappa), 0.209 (Delta), and 2.768 (Omicron) mg mL^−1^ (Figure 3b). Compared with the pseudotyped SARS‐CoV‐2 Wuhan‐1 strain, the neutralizing titer of COVID‐HIG was generally similar to that of the pseudotyped D614G mutant (1.1‐fold), Alpha (–1.3‐fold), and Gamma (–1.6‐fold) variants; but decreased against the pseudotyped Beta (–7.3‐fold), Kappa (–5.0‐fold), Delta (–2.6‐fold), and Omicron (–35.0‐fold) variants (Figure 3c). In addition, the neutralization potency of COVID‐HIG based on the pseudotyped SARS‐CoV‐2 Wuhan‐1 strain was ≈4.2‐fold higher than that of PBP (Table S4, Supporting Information). In contrast, IVIG displayed no neutralization effect against Wuhan‐1, Beta, or Delta variants (Figure S1, Supporting Information).

### 2.4 COVID‐HIG Neutralizes Authentic SARS‐CoV‐2 Beta and Delta Variants

The above results of PBNAs showed that the neutralization activity of COVID‐HIG against the Beta variant was substantially lower than that against the other tested variants (Figure 3), and the Delta variant has become one of the most worrisome strains of SARS‐CoV‐2 circulating globally. Therefore, we used authentic Beta and Delta variants for further testing.

**Figure 4:**
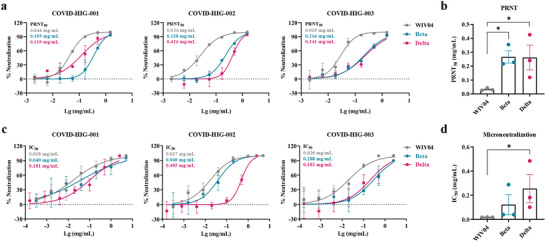
Neutralization of COVID‐HIG against SARS‐CoV‐2 WIV04, Beta, and Delta strains in vitro. a) PRNTs showed that COVID‐HIG‐001, ‐002, and ‐003 significantly inhibited infection by SARS‐CoV‐2 WIV04, Beta, and Delta strains in Vero E6 cells. Viruses were incubated with COVID‐HIG at 37 °C for 1 h. Next, Vero E6 cells were infected with WIV04, Beta, and Delta strains and stained with hematoxylin/eosin at 48 h (Beta and Delta variants) or 72 h (WIV04 strain) postinfection. The *y*‐axis represents percent inhibition. The mean from two independent replicates is shown (*n* = 2). b) Comparison of the PRNT_50_ of the variants and WIV04; statistical significance was analyzed using one‐way ANOVA. *P < 0.05. c) Microneutralization assays showed that COVID‐HIG‐001, ‐002, and ‐003 significantly inhibited the SARS‐CoV‐2 WIV04, Beta, and Delta strains in Vero E6 cells. Viruses were incubated with COVID‐HIG at 37 °C for 1 h. Next, Vero E6 cells were infected with the WIV04, Beta, and Delta strains. After 24 h, the infected cell supernatant was analyzed using real‐time reverse transcription‐PCR (qRT‐PCR). The *y*‐axis represents percent inhibition. The mean from two independent replicates is shown (*n* = 2). d) Comparison of the IC_50_ of the variants and WIV04; statistical significance was analyzed using one‐way ANOVA. *P < 0.05. PRNT, plaque reduction neutralization tests.

Plaque reduction neutralization tests (PRNTs) showed that all three batches of COVID‐HIG significantly reduced plaque formation after infection with the SARS‐CoV‐2 WIV04, Beta, and Delta strains (**Figure** **4**a and Figure S2, Supporting Information). The average 50% plaque reduction neutralization test concentration (PRNT_50_) of the three batches of COVID‐HIG against these three strains were 0.033, 0.267, and 0.262 mg mL^−1^, respectively (Figure 4a,b). Cell‐level microneutralization tests also showed that all three batches of COVID‐HIG clearly inhibited the SARS‐CoV‐2 WIV04, Beta, and Delta strains (Figure 4c). The average IC_50_ values of COVID‐HIG against these three strains were 0.018, 0.123, and 0.256 mg mL^−1^, respectively (Figure 4c,d). The results obtained using both the plaque reduction neutralization and microneutralization methods showed that COVID‐HIG was more effective in neutralizing the WIV04 strain than the Beta or Delta variants. Compared with that against the WIV04 strain, the PRNT_50_ values of COVID‐HIG were significantly decreased against the Beta (8.1‐fold) and Delta (8.0‐fold) variants; additionally, the IC_50_ values of COVID‐HIG were decreased against the Beta (6.8‐fold) and Delta (14.1‐fold) variants (Figure 4b,d).

### 2.5 Prophylactic Treatment with COVID‐HIG Effectively Protects Adv5‐hACE2‐Transduced Mice against SARS‐CoV‐2 Infection

Previous studies have shown that SARS‐CoV‐2 successfully infected replication‐deficient adenovirus (Adv5)‐hACE2‐transduced mice in which type I interferon receptors had been knocked out (IFNAR^−/−^), resulting in weight loss, a high viral load, and severe lesions in the lungs. After appropriate therapeutic interventions, the changes in both the viral load and pathological damage in the lungs were reversed.^[^ ^27^, ^28^ ^]^ Therefore, the antiviral activity of COVID‐HIG (COVID‐HIG‐002) against SARS‐CoV‐2 infection in vivo was tested in this mouse model. The dose used was referenced to that of anti‐coronavirus hyperimmune intravenous immunoglobulin dose used in clinical trials to treat hospitalized patients with COVID‐19 (400 mg kg^−1^; https://www.niaid.nih.gov/news‐events/nih‐clinical‐trial‐testing‐hyperimmune‐intravenous‐immunoglobulin‐plus‐remdesivir‐treat). In addition, maltose, the pharmaceutical excipient of COVID‐HIG, was used as a control.

**Figure 5:**
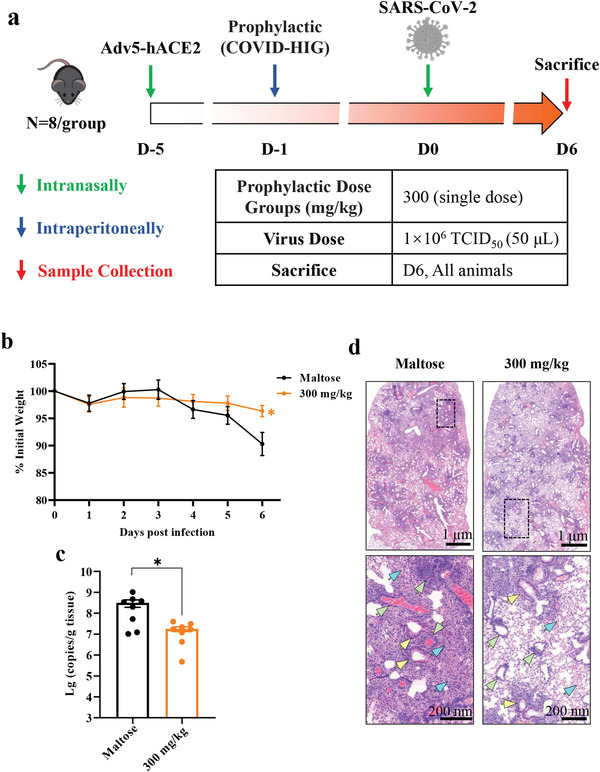
Prophylactic treatment with COVID‐HIG protects mice from SARS‐CoV‐2 infection. a) Experimental design. Adv5‐hACE2 was intranasally inoculated into IFNAR^−/−^ C57BL/6 mice (*n* = 8 per group). Five days after transduction, Adv5‐hACE2‐transduced mice were intraperitoneally injected with 300 mg kg^−1^ COVID‐HIG 24 h before (prophylactic treatment) infection or with 200 µL 10% maltose 2 h after SARS‐CoV‐2 infection (control, shared with the therapeutic treatment group). b) Daily body weight changes in COVID‐HIG prophylactic‐treated or maltose‐treated mice. Data are shown as the mean ± SEM of *n* = 8 animals per group. Statistical significance was determined using two‐way ANOVA. **P < 0.01. c) Viral RNA levels in the lung tissues of COVID‐HIG prophylactic‐treated or maltose‐treated mice were determined using qRT‐PCR at 6 day‐ post‐infection. Data are represented as mean ± SEM of *n* = 8 animals per group. Statistical significance was determined using an independent *t*‐test. *P < 0.05. d) Histopathological analyses of COVID‐HIG‐treated or untreated mice challenged with SARS‐CoV‐2. Representative images of lung sections stained with hematoxylin and eosin at 6 day‐post‐challenge. Blue arrows indicate pathological changes in the alveolar wall and alveolar cavity. Yellow arrows indicate bronchiole lesions. Green arrows indicate pathological changes to blood vessels. The image in the lower panel is an enlarged view of the black dotted box in the image in the upper panel.

First, we tested the prophylactic effect of COVID‐HIG. The mice were intranasally transduced with 4 × 10^8^ of the median tissue culture infective dose (TCID_50_) of Adv5‐hACE2 to induce hACE2 expression in the lungs. At 5 day‐post‐transduction, the animals were challenged with 1 × 10^6^ TCID_50_ SARS‐CoV‐2 WIV04 strain via the intranasal route and monitored for 6 days. For prophylactic treatment, the mice were intraperitoneally injected with 300 mg kg^−1^ COVID‐HIG 24 h prior to SARS‐CoV‐2 infection. The control mice were injected with 200 µL maltose (10%) at 2 h post SARS‐CoV‐2 infection (**Figure** **5**a). Severe weight loss was detected in the maltose control group, whereas mice pretreated with COVID‐HIG showed only slight reduction in body weight (*P *< 0.01) (Figure 5b). The viral load in the lungs was reduced by more than tenfold (*P *< 0.05) after COVID‐HIG treatment (Figure 5c). Hematoxylin and eosin staining displayed severe pathological injury, diffuse alveolar injury, extensive inflammatory cell infiltration, hyaline membrane formation, and fibrosis in lung samples from the control group, with an average pathological score of 5.0 (Figure 5d and Table S5, Supporting Information). In contrast, COVID‐HIG treatment significantly alleviated virus‐induced lung injury, with an average pathological score of 2.8 (Figure 5d and Table S5, Supporting Information). Observation of the gross anatomy also indicated that the pulmonary pathological changes in the control group were more severe than in the treatment group (Figure S3, Supporting Information). Furthermore, immunofluorescence staining of SARS‐CoV‐2 nucleocapsid proteins demonstrated that COVID‐HIG treatment effectively reduced the viral load in lung samples (Figure S4, Supporting Information). Thus, prophylactic treatment with COVID‐HIG significantly improved body weight loss, reduced the pulmonary viral load, and alleviated pathological lung injury in SARS‐CoV‐2‐infected mice.

### 2.6 COVID‐HIG Shows In Vivo Therapeutical Efficacy against SARS‐CoV‐2 Infection in a Dose‐Dependent Manner

**Figure 6:**
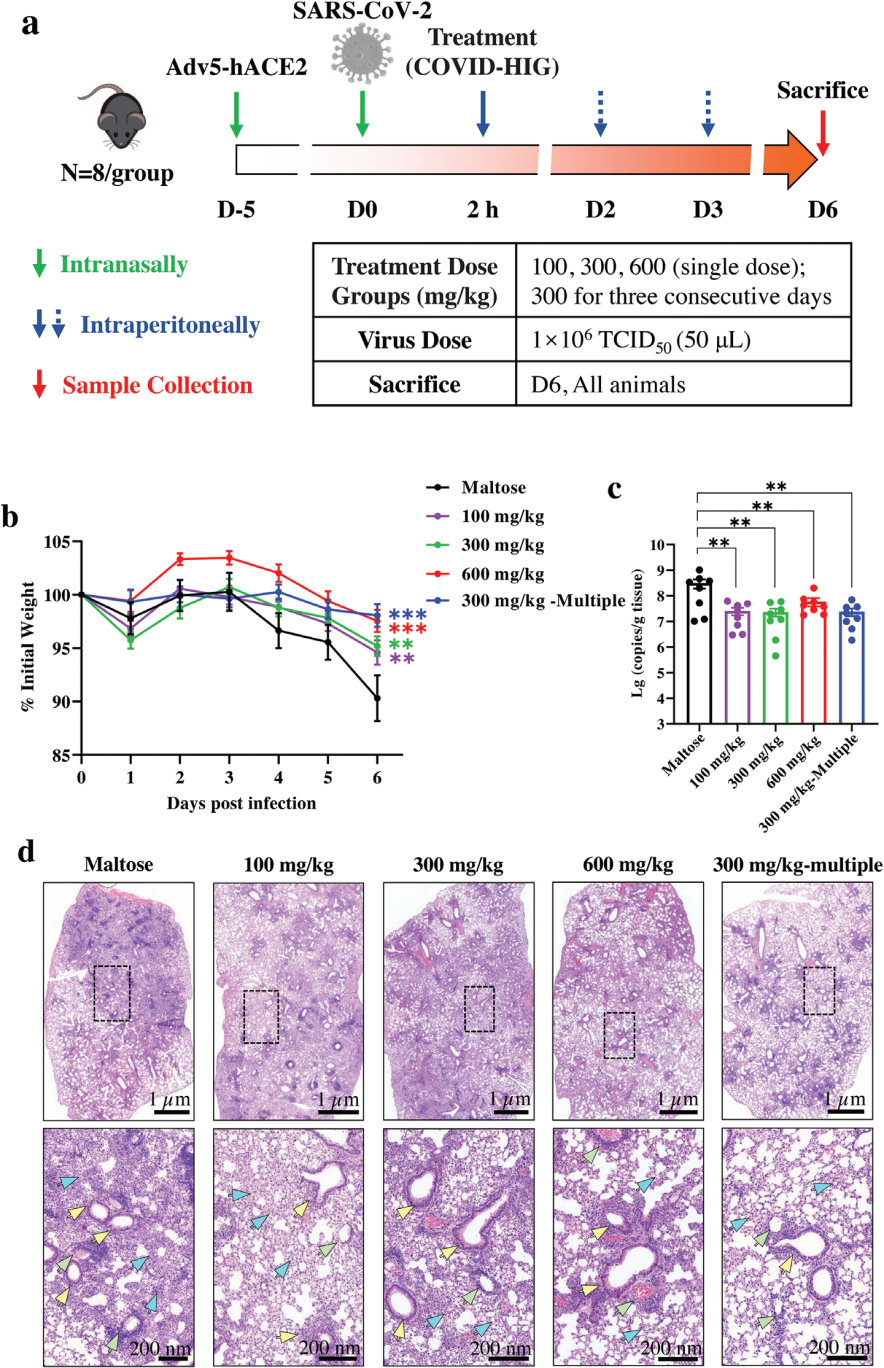
Therapeutic treatment with COVID‐HIG protects mice from SARS‐CoV‐2 infection. a) Experimental design. Adv5‐hACE2‐transduced mice were intraperitoneally injected once with 600, 300, or 100 mg kg^−1^ COVID‐HIG 2 h after SARS‐CoV‐2 infection (*n* = 8 per group). Another group was injected with 300 mg kg^−1^ COVID‐HIG 0, 1, and 2 d after SARS‐CoV‐2 infection (therapeutic treatment, *n* = 8 per group). The control group was injected with 200 µL 10% maltose 2 h after (control) SARS‐CoV‐2 infection (*n* = 8 per group). b) Daily body weight changes of COVID‐HIG therapeutic group or maltose‐treated mice. Data are shown as the mean ± SEM of *n* = 8 animals per group. Statistical significance was determined using two‐way ANOVA. *P < 0.05, **P < 0.01, ***P < 0.001. c) Viral RNA levels in the lung tissues of the COVID‐HIG therapeutic group or maltose‐treated mice were determined using qRT‐PCR at 6 day‐post‐infection. Data are represented as the mean ± SEM of *n* = 8 animals per group. Statistical significance was determined using one‐way ANOVA. **P < 0.01, ***P < 0.001. d) Histopathological analyses of COVID‐HIG‐treated or untreated mice challenged with SARS‐CoV‐2. Representative images of lung sections stained with hematoxylin and eosin at 6 day‐post‐challenge. Blue arrows indicate pathological changes to the alveolar wall and alveolar cavity. Yellow arrows indicate bronchiole lesions. Green arrows indicate pathological changes to the blood vessels. The image in the lower panel is an enlarged view of the black dotted box in the image in the upper panel.

Simultaneously, the therapeutic efficacy of COVID‐HIG was tested with different doses (single doses: 100, 300, or 600 mg kg^−1^; multiple dose: 300 mg kg^−1^ for three consecutive days) (**Figure** **6**a). For the single‐dose groups, 600 mg kg^−1^ treatment was more effective (*P *< 0.001) in rescuing body weight loss compared to 300 and 100 mg kg^−1^ treatment groups (*P *< 0.01) (Figure 6b). The multiple‐dose group showed only slight body weight loss compared to the maltose control group (*P *< 0.001) (Figure 6b). For all four therapeutic groups, the viral loads were significantly reduced by more than tenfold (Figure 6c). Similarly, all COVID‐HIG therapeutic groups displayed less pathological injury of the lungs. The 300 mg kg^−1^ multiple‐dose group showed the best therapeutic effect, followed by the 600 mg kg^−1^ single‐dose group (Figure 6d). The average pathological scores of the 300 mg kg^−1^ multiple and 600, 300, and 100 mg kg^−1^ groups were 2.9, 3.2, 3.9, and 3.6, respectively (Table S5, Supporting Information). The treatment effects were also evaluated by pulmonary gross anatomy observation and immunofluorescence staining (Figures S3 and S4, Supporting Information). Thus, COVID‐HIG therapeutic treatment inhibited SARS‐CoV‐2 infection in vivo in a dose‐dependent manner, and multiple‐dose treatment (300 mg kg^−1^) had an obvious advantage over a single middle (300 mg kg^−1^)‐ or low (100 mg kg^−1^)‐dose treatment.

### 2.7 Comparison of RBD‐IgG Titers and Neutralization Potency between COVID‐HIG and Pooled Convalescent Plasma (PCP)‐HIG

We also prepared three batches of anti‐SARS‐CoV‐2 hyperimmune globulin from PCP of patients recovered from COVID‐19 (hereafter referred to as PCP‐HIG) in a similar manner as used for COVID‐HIG. The final batch numbers were PCP‐HIG‐001 (217 combined plasma samples), PCP‐HIG‐002 (226 combined plasma samples), and PCP‐HIG‐003 (245 combined plasma samples). RBD‐IgG titers and the neutralization potency of COVID‐HIG and PCP‐HIG were measured to compare the differences between the two types of anti‐SARS‐CoV‐2 hyperimmune globulins.

**Figure 7:**
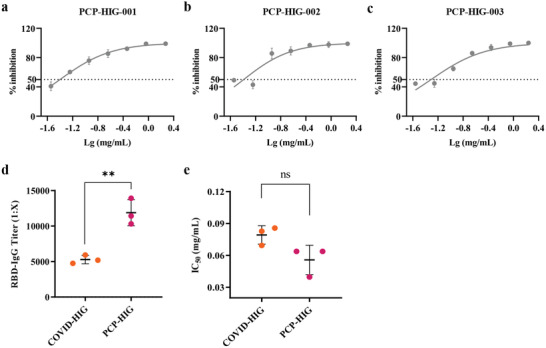
Comparison of RBD‐IgG titer and neutralization potency between COVID‐HIG and PCP‐HIG. Three batches of PCP‐HIG— a) PCP‐HIG‐001, b) PCP‐HIG‐002, and c) PCP‐HIG‐003 —have potent neutralizing activities against the pseudotyped SARS‐CoV‐2 Wuhan‐1 strain. Data are shown as mean ± SD from three independent experiments (*n* = 3). d) RBD‐IgG titers of COVID‐HIG and PCP‐HIG were determined using SARS‐CoV‐2 RBD ELISA. The mean ± SD from three independent experiments (*n* = 3) of COVID‐HIG (COVID‐HIG‐001, ‐002, and ‐003) or PCP‐HIG (PCP‐HIG‐001, ‐002, and ‐003) is shown. Statistical significance was determined using *t*‐test. **P < 0.01. e) Neutralization potency of COVID‐HIG and PCP‐HIG against the pseudotyped SARS‐CoV‐2 Wuhan‐1 strain. The mean ± SD of COVID‐HIG (COVID‐HIG‐001, ‐002, and ‐003) or PCP‐HIG (PCP‐HIG‐001, ‐002, and ‐003) is shown. Statistical significance was determined using *t*‐test. ns, no significance (*P >* 0.05). PCP‐HIG, anti‐SARS‐CoV‐2 hyperimmune globulin from pooled convalescent plasma of donors who had recently recovered from COVID‐19. ELISA, enzyme‐linked immunosorbent assay. PBNAs, pseudotyped virus‐based neutralization assays.

The RBD‐IgG titers of COVID‐HIG and PCP‐HIG were determined using SARS‐CoV‐2 RBD ELISA. Additionally, changes in the RBD‐IgG titers between the three batches of COVID‐HIG and their corresponding PBP were compared. The average value of the COVID‐HIG RBD‐IgG titer was 7.3‐fold higher than that of PBP (Table S6, Supporting Information). PBNAs, evaluated using the same detection methods as applied for COVID‐HIG, were also used to confirm the neutralization potency against the pseudotyped SARS‐CoV‐2 Wuhan‐1 strain of PCP‐HIG. All three batches of PCP‐HIG (PCP‐HIG‐001, ‐002, and ‐003) showed a clear inhibitory effect on the SARS‐CoV‐2 Wuhan‐1 strain with IC_50_ values of 0.040 (PCP‐HIG‐001), 0.064 (PCP‐HIG‐002), and 0.064 (PCP‐HIG‐003) mg mL^−1^, respectively (**Figure** **7**a–c). We next compared the difference in the RBD‐IgG titers between COVID‐HIG and PCP‐HIG. The RBD‐IgG titer of COVID‐HIG was significantly (*P *= 0.0042) lower than that of PCP‐HIG, showing an ≈2.2‐fold difference (Figure 7d). Notably, there was no significant difference (*P *= 0.068) in neutralization potency against the Wuhan‐1 strain between COVID‐HIG and PCP‐HIG (Figure 7e).

## 3 Conclusion

The plasma of healthy individuals vaccinated with BBIBP‐CorV contains anti‐SARS‐CoV‐2 antibodies with neutralizing activity against various mutant variants such as Alpha and Beta.^[^ ^14^, ^15^ ^]^ However, there are some limitations to using vaccine‐immunized plasma, such as large differences in the neutralizing antibody titer, potential risk from blood‐borne viruses, need for blood cross‐matching, and difficulty in storage and transportation. Therefore, we produced COVID‐HIG from PBP of vaccine‐immunized donors (Figure 1). The IgG concentration (5.5‐fold), RBD‐IgG titer (7.3‐fold), and neutralization potency based on the pseudotyped SARS‐CoV‐2 Wuhan‐1 strain (4.2‐fold) were markedly increased when the PBP was processed into COVID‐HIG (Tables S1, S4, and S6, Supporting Information). This suggests that patients with COVID‐19 treated with COVID‐HIG can be administered a much smaller intravenous volume, which may decrease the risk of transfusion‐associated circulatory overload.

Genetic variation in SARS‐CoV‐2 has appeared throughout the COVID‐19 pandemic and has spread worldwide. Some SARS‐CoV‐2 variants may increase disease severity and even lead to higher mortality.^[^ ^29^, ^30^ ^]^ SARS‐CoV‐2 containing the D614G mutation has caused fatal infections in many European countries,^[^ ^31^ ^]^ with previous studies suggesting that the increase in mortality is related to this mutation.^[^ ^31^, ^32^ ^]^ In Britain, the Alpha variant may be more easily transmitted between people compared with preexisting SARS‐CoV‐2 variants.^[^ ^2^ ^]^ The Beta variant spread rapidly and became dominant in South Africa within weeks.^[^ ^3^ ^]^ Gamma was also purported to be highly transmissible.^[^ ^4^, ^5^ ^]^ The Kappa and Delta variants show particularly high transmissibility, raising concerns among public health experts.^[^ ^6^ ^]^ The recently emerged Omicron variant cause substantial immune evasion from a panel of existing neutralizing monoclonal antibodies.^[^ ^33^ ^]^ These seven pseudotyped SARS‐CoV‐2 variants of concern or interest containing key mutations were used to detect whether these mutations allow the virus to escape COVID‐HIG activity. The results clearly showed that COVID‐HIG inhibited all pseudotyped SARS‐CoV‐2 variants in vitro (Figure 3). Authentic viruses were then used to verify the antiviral neutralizing activity of COVID‐HIG in vitro. The neutralization potency of COVID‐HIG against the Beta or Delta variant was lower than that against the WIV04 strain, but these two authentic variants were still effectively neutralized (Figure 4). COVID‐HIG contains versatile anti‐SARS‐CoV‐2 antibodies (a pool of different monoclonal antibodies), and shows high‐affinity binding to the full‐length S protein, RBD, NTD, and NP (Figure 2a,b). Some antibodies from healthy donors immunized with BBIBP‐CorV still retain the ability to bind various mutant virus strains,^[^ ^15^ ^]^ explaining why COVID‐HIG has a neutralizing effect on various SARS‐CoV‐2 mutant strains.

COVID‐HIG was found to have multiple high‐affinity epitope‐binding and hACE2‐blocking properties, which translated to high neutralization potency in vitro and potent prophylactic and treatment efficacy in vivo. Potency refers to the concentration (IC_50_ or PRNT_50_) of COVID‐HIG required to produce 50% of the maximum effect. Efficacy is the maximum effect which can be expected from COVID‐HIG. Several mechanisms of neutralization of SARS‐CoV‐2 antibodies have been proposed, including a) antibodies bind to the RBD of S protein and compete with hACE2 binding; b) antibodies bind to the RBD (excluding the receptor‐binding motif), the NTD or subunit 2 of the S protein, but do not compete for hACE2 binding; and c) antibodies (or cocktails) bind to multiple epitopes and neutralize virus by blocking RBD binding or restricting conformational changes in the S protein.^[^ ^34^ ^]^ Neutralization of COVID‐HIG, as discussed here, is defined as the reduction in viral infectivity by the binding of antibodies to SARS‐CoV‐2, thereby blocking any step in the viral replication cycle before the virally encoded transcription or synthesis event.^[^ ^35^ ^]^ In this study, we proved that COVID‐HIG antibodies could effectively bind RBD and block the binding of the SARS‐CoV‐2 RBD to hACE2 with high ability, ultimately inhibiting viral infection (Figure 2c,d). Additionally, COVID‐HIG antibodies binding to multiple epitopes of the S protein, the RBD (excluding the receptor‐binding motif), the NTD or the NP may lead to neutralization activity against SARS‐CoV‐2 through restricting conformational changes or via unknown multiple mechanisms. COVID‐HIG showed higher affinity to the Beta and Delta RBDs than that to WIV04 RBD (Figure 2c and Table S3, Supporting Information). Higher affinity means greater potent binding to the Beta and Delta RBDs, but not greater neutralization potency toward the Beta and Delta variants (Figure 4 and Table S3, Supporting Information). This discrepancy could be due to the potential synergistic neutralizing effects of the antibody cocktails in COVID‐HIG, and the RBD affinity data cannot fully represent the neutralizing ability of COVID‐HIG. We also estimated the COVID‐HIG neutralization efficiency for different epitopes by calculating the *K*_D_/PRNT_50_ ratio (Table S3, Supporting Information), the different ratios indicate the existence of epitope‐specific differences in neutralization efficiency.^[^ ^36^ ^]^

Next, we evaluated the in vivo efficacy of COVID‐HIG in SARS‐CoV‐2‐infected hACE2‐Adv5‐transduced IFNAR^−/−^ mice. The COVID‐HIG ingredients comprise highly purified IgG (≥97% purity) and 10% maltose; IgG contains anti‐SARS‐CoV‐2 antibodies as the active ingredient of COVID‐HIG. We used maltose, a pharmaceutical excipient of COVID‐HIG, as a control. Prophylactic and therapeutic application of COVID‐HIG alleviated body weight loss, reduced the number of virus copies in the lungs, and alleviated pathological damage to the lungs (Figures 5 and 6). Data from randomized trials to reliably assess the safety and efficiency of CP remain lacking. In January and March of 2021, the Mayo Clinic^[^ ^20^ ^]^ and the RECOVERY Collaboration Group^[^ ^21^ ^]^ published two clinical results of using CP to treat patients with COVID‐19. Both studies included hospitalized patients with COVID‐19 who did not receive mechanical ventilation and were treated with high‐titer CP. The mortality rates within 30 or 28 d were determined, but the results of the two trials were inconsistent. Many patients with COVID‐19 have been treated with CP, but the dosage and frequency of this treatment depends on experience. Most CP protocols adopt a single infusion without exploring the dosage and usage through nonclinical efficacy.^[^ ^18^, ^19^, ^20^, ^21^ ^]^ In mice, treatment with multiple doses of COVID‐HIG showed better protective effects compared to a single dose (Figure 5). In addition, prophylactic administration at the same dose (300 mg kg^−1^ COVID‐HIG) was more effective than therapeutic administration (Figure S3 and Table S5, Supporting Information). COVID‐HIG showed strong anti‐SARS‐CoV‐2 activity in our mouse model, which should enhance patient confidence in using passive immunotherapy for COVID‐19 and provides a useful reference for subsequent clinical research of COVID‐HIG. Previously, most nonclinical studies of preventive or therapeutic COVID‐19 drugs focused on monoclonal antibodies or chemical drugs.^[^ ^37^, ^38^, ^39^, ^40^ ^]^ Few studies have examined the efficacy of CP or anti‐SARS‐CoV‐2 hyperimmune globulin in vivo. Thus, this study was performed to fill this knowledge gap.

Moreover, our study showed that although the RBD titer of COVID‐HIG was much lower than that of PCP‐HIG, their neutralization potencies were similar (Figure 7d,e). Regarding the production of anti‐SARS‐CoV‐2 hyperimmune globulin using PCP as the raw material, the number of people vaccinated against COVID‐19 has greatly exceeded the number of people infected with SARS‐CoV‐2. According to statistics of the World Health Organization (https://covid19.who.int/), the number of vaccines is far greater than that of people infected. Therefore, the number of potential PBP donors is much higher than that of PCP donors. In addition, when PBP is used as the raw material to produce anti‐SARS‐CoV‐2 hyperimmune globulin, there is no risk of SARS‐CoV‐2 exposure during the process of plasma collection and product preparation. Additionally, although CP is obtained from patients infected with SARS‐CoV‐2, it is necessary to consider that near‐sourced CP likely reflects the antigenic composition of local viral strains, which may be the reason for the different clinical results of CP. Kunze et al.^[^ ^41^ ^]^ showed the mortality of CP treatment varies depending on the geographic origin of donors. Further, clinical research results of CP therapy remain controversial.^[^ ^19^, ^20^, ^21^ ^]^ In this study, plasma was collected from healthy donors who had been vaccinated with BBIBP‐CorV; thus, the different donors contained the same antigens. Thus, research on the effectiveness of plasma after vaccination should have higher replicability.

There are also limitations in the production and clinical application of COVID‐HIG. The pharmacokinetics of COVID‐HIG and whether the hyperimmune globulin will induce an immune response have not been tested. IVIG may lead to protection during an infection in vivo via Fc dependent actions,^[^ ^42^ ^]^ and should be more suitable as a control in animal experiments. Unlike some oral drugs, hyperimmune globulin products can only be administered intravenously or subcutaneously in the clinic. In addition, COVID‐HIG needs to be prepared with human plasma as the raw material, and the collection of plasma may be associated with unstable factors. The efficacy of most mRNA or recombinant vaccines and monoclonal antibodies is based on anti‐SARS‐CoV‐2 S neutralizing activity, hyperimmune globulin therapy is somewhat similar to monoclonal antibody therapy. However, the targets of COVID‐HIG include the RBD, NTD, and NP. Compared with monoclonal antibodies, COVID‐HIG derived from inactivated vaccines has more anti‐SARS‐CoV‐2 targets and should therefore have stronger ability to resist the immune escape of SARS‐CoV‐2 variants.

In conclusion, COVID‐HIG largely neutralized various SARS‐CoV‐2 mutant viruses and showed promising preventive and therapeutic effects in a mouse model infected with SARS‐CoV‐2, supporting its potential as a clinical treatment strategy. Moreover, our results can improve confidence in the use of hyperimmune globulin, the traditional passive immunotherapy, for treating COVID‐19. We believe that COVID‐HIG might provide an alternative option to combat COVID‐19, which is currently being evaluated in clinical trials (clinicaltrials.gov NCT05173441).

## 4 Experimental Section

### Donor Immunization

The basic immunization method used the Sinopharm COVID‐19 Vaccine (Vero Cell‐inactivated) (also known as BBIBP‐CorV) produced by Beijing Institute of Biological Products Co., Ltd (Beijing, China). Immunization was performed using the recommended two‐dose procedure with a 28 d interval.

### Donor Screening and Plasma Collection

*Immunized Healthy Donors for Plasma Collection*: Plasma containing anti‐SARS‐CoV‐2 antibody was collected from immunized healthy donors (immunization described above). All donors were screened for transfusion‐transmitted infections (human immunodeficiency virus, hepatitis‐B, hepatitis‐C, and syphilis spirochete). The results of serology screening were all negative. Immunized healthy donors who had been infected with SARS‐CoV‐2 or SARS‐CoV‐1 were excluded.

*COVID‐19 Convalescent Donors for Convalescent Plasma Collection*: Plasma was collected from donors who had recovered from COVID‐19, and met the “Diagnosis and Treatment Protocol for Novel Coronavirus Pneumonia (Trial Version 4 and subsequent versions),” released by the National Health Commission and State Administration of Traditional Chinese Medicine. The donors were screened by clinicians following blood donation standards. The donor's plasma was collected within three months after recovery and the convalescent patients included those with mild, moderate, and severe disease courses.

Plasma Collection: The requirements for source plasma (no clots, no fibrin precipitation, fat‐free blood, and hemolysis) were confirmed. The collected plasma was stored at −20 °C or below. The storage period should not exceed 3 years from the date of plasma collection. Plasma donors were able to donate up to 600 g (plasma donated by each person at each time was used as a single plasma sample), and the interval between two plasma collections was no less than 14 days.

This study was approved by the Ethics Committee of Tiantan Biological R&D Center (approval numbers KY‐2020EC‐01 and KY‐2020EC‐02), and each participant signed an informed consent statement.

### COVID‐HIG Production

The commercial IVIG production method (cold ethanol fractionation) with some modifications, was used to produce COVID‐HIG.^[^ ^43^, ^44^, ^45^ ^]^ Pooled plasma was prepared for subsequent processing by thawing at less than 37 °C. The anti‐SARS‐CoV‐2 antibody titer of the pooled plasma was measured using ELISA before processing.

Pooled plasma was subjected to ethanol fractionation, pressure filtration, and purification using a 30–50 kDa ultrafiltration membrane (Sartorius, Göttingen, Germany). This process concentrated the immunoglobulins and removed impurities, resulting in highly purified‐bulk IgG (97% and above of total proteins), with 10% maltose for stabilization. The production process, including formulation, was identical to that used to prepare IVIG (pH 4) for commercial use, resulting in highly purified IgG solutions formulated with 5% protein and 10% maltose at a low pH (pH 4).

The bulk IgG was passed through sterile 0.2‐µm filters and collected into pyrogen‐free containers. Next, the potential viruses were inactivated and removed by low pH (pH 3.8–4.4) incubation at 24 ± 1 °C for 21 days, followed by nanofiltration through a 50 nm filter membrane (Ultipor DV50 Cartridges, Pall, Port Washington, NY, USA), yielding the final COVID‐HIG product.

### Detection of SARS‐CoV‐2 RBD‐IgG Titer

A Conformité Européene‐marked coronavirus IgG antibody detection kit (product code: WS‐1396) from Beijing WanTai Biological Pharmacy Enterprise Co., Ltd. (Beijing, China) was used to test the RBD‐IgG titer. The kit employs a solid phase, indirect ELISA method to detect IgG‐class antibodies to SARS‐CoV‐2 in a two‐step incubation procedure. The RBD‐IgG titer was calculated using a four‐parameter equation curve fitted to the measured optical density and standard concentration. ELISA was performed as previously described.^[^ ^46^ ^]^

### Cells

Vero E6 (American Type Culture Collection, Manassas, VA, USA; no. 1586) and Huh‐7 (National Collection of Authenticated Cell Cultures, Shanghai, China; TCHu182) cells were maintained in minimum Eagle's medium (Gibco) and Dulbecco's modified Eagle medium (Gibco, Grand Island, NY, USA), respectively. The medium was supplemented with 10% fetal bovine serum (Gibco). The cells were cultured at 37 °C in 5% CO_2_.

### FACS Assay

Binding tests of COVID‐HIG or IVIG (lot number 201904009; Sinopharm Wuhan Plasma‐derived Biotherapies Co., Ltd, Wuhan, China) to CHO‐K1/Spike cells (Genscript, Nanjing, China) were assessed by FACS. CHO‐K1/Spike cells stably overexpressed the SARS‐CoV‐2 S protein (National Center for Biotechnology Information Reference Sequence: YP_009724390.1). COVID‐HIG or IVIG (starting from 1 mg mL^−1^, six concentrations with a dilution factor of five) was mixed with 3 × 10^5^ CHO‐K1/Spike cells and incubated at 4 °C for 1 h. The cells were washed with Dulbecco's phosphate‐buffered saline twice and mixed with anti‐Human IgG (H+L) secondary antibody (Alexa Fluor 647, Thermo Fisher, Scientific, Waltham, MA, USA) at a concentration of 4 µg mL^−1^. The mixtures were incubated for 1 h at 4 °C and washed with Dulbecco's phosphate‐buffered saline twice. The cells were resuspended and detected using flow cytometry (NovoCyte 3005, ACEA Biosciences, CA, San Diego, USA).

### BLI Measurement of Affinity and Competition‐Binding Study

The S protein, the NP of SARS‐CoV‐2 WIV04 strain, and the RBDs of WIV04, Beta and Delta were expressed in 293‐FT cells. The NTD was purchased from Sino Biological Inc. (Cat. No. 40591‐V49H). The binding of COVID‐HIG (COVID‐HIG‐003) to the recombinant S protein, NTD, NP, and RBDs was analyzed using BLI with an Octet‐Red 96 device (Pall ForteBio LLC., CA, USA). All steps were performed in a black 96‐well plate with a working volume of 200 µL per well at 30 °C, with shaking at 400 rpm. By using RBDs as examples, 10 µg purified RBDs labeled with biotin per well were loaded onto streptavidin biosensors (ForteBio), which were activated in binding buffer (0.1% w/v bovine Serum Albumin (BSA), 0.01% w/v Tween‐20 in phosphate‐buffered saline) for 300 s. The sensors were then dipped into the serially diluted COVID‐HIG (0.16–20 *μ*M) for 600 s for measurement of association kinetics after incubation for 180 s in the baseline buffer (0.1% w/v BSA, 0.01% w/v Tween 20 in PBS). Then, the dissociation was measured in a kinetic buffer (0.1% w/v BSA, 0.01% w/v Tween 20 in PBS) for 600 s. Octet Data Acquisition 9.0 was used for data analysis and curve fitting using an 1:1 model. The *K*_D_ (the ratio of *k*_dis_ to *k*_on_), which is the equilibrium dissociation constant between antibodies and antigens, was calculated to represent affinities of different RBDs with COVID‐HIG affinity. The BLI assays of the S protein, NTD and NP with COVID‐HIG were performed using similar methods.

For the ELISA competition‐binding assays, RBDs fused with S‐tag were immobilized on the chips. 50 µL of serially diluted COVID‐HIG (COVID‐HIG‐003; 0.0156–2 mg or 0.062–4 mg) and 50 µL dilution buffer as a negative control to the chips, followed by 50 µL ACE2 protein conjugated with horseradish peroxidase were added. After incubation at 37 °C for 30 min, 3,3’,5,5’‐tetramethylbenzidine substrate was added and incubated at 37 °C for 15 min. The OD_450_ values were then determined after the reaction was terminated. Inhibition (%) = (1‐ sample OD_450_/negative control OD_450_) (%), and the IC_50_ values were determined using nonlinear regression analysis.

### Pseudotyped Viruses‐Based SARS‐CoV‐2 Neutralizing Antibody Assay

Pseudotyped viruses were obtained from Gobond Science and Technology (Beijing) Co., Ltd (Beijing, China). Specific information is shown in Table S7 in the Supporting Information. The pseudotyped virus‐based neutralization test used in this study was developed by the National Institutes for Food and Drug Control.^[^ ^26^ ^]^ Experimental samples (PBP, COVID‐HIG, and PCP‐HIG) were serially diluted by twofold in Dulbecco's modified Eagle medium (Gibco) and incubated with pseudotyped viruses (≈1.3 × 10^4^ TCID_50_ mL^−1^) for 1 h at 37 °C. Freshly trypsinized Huh‐7 cells (2 × 10^4^) were added to each well. Following 24‐h incubation in a 5% CO_2_ environment at 37 °C, luciferase substrate (PerkinElmer, Waltham, MA, USA) was added to each well. The samples were incubated at room temperature for 2 min and luminescence was detected using a microplate luminometer (GloMax Navigator, Promega, Madison, WI, USA). The luminescence of pseudotyped virus + Huh‐7 cell wells was used as the virus control, that of Huh‐7 cells only was used as the background control; and that of experimental samples (PBP, COVID‐HIG, and PCP‐HIG) + pseudotyped virus + Huh‐7 cell wells was the experimental group. The % neutralization = [1 – (experimental group – background control)/(virus control – background control)] × 100%.

### Viruses

The SARS‐CoV‐2 WIV04 strain (nCoV‐2019BetaCoV/Wuhan/WIV04/2019)^[^ ^25^ ^]^ was stored at the National Virus Resource Center. The SARS‐CoV‐2 Beta variant, National Pathogen Resource Center (NPRC) 2.062100001;^[^ ^47^ ^]^ and the SARS‐CoV‐2 Delta variant (China Science and Technology Resource. 16698.06.NPRC 6. CCPM‐B‐V‐049‐2105‐8), provided by the National Pathogen Resource Center were propagated in Vero E6 cells. The viral titer (TCID_50_ mL^−1^) was determined using indirect immunofluorescence assay with Vero E6 cells. Adv5‐hACE2 was constructed and amplified as previously described.^[^ ^48^ ^]^ All studies related to infectious SARS‐CoV‐2 were conducted in a biosafety level‐3 laboratory.

### Plaque Reduction Neutralization Test

Vero E6 cells (1.2 × 10^5^) were seeded into 24‐well plates and cultured overnight, after which COVID‐HIG‐001, COVID‐HIG‐002, and COVID‐HIG‐003 were serially diluted by threefold (maximum concentration, 5 mg mL^−1^) in minimum Eagle's medium and incubated with SARS‐CoV‐2 WIV04, SARS‐CoV‐2 Beta, or SARS‐CoV‐2 Delta (41000 TCID_50_ mL^−1^) for 1 h at 37 °C; Maltose (0.1%) was used as a negative control. The immunoglobulin‐virus mixture was added to Vero E6 cells in duplicate and incubated for 1 h at 37 °C in 5% CO_2_. The immunoglobulin‐virus mixture was removed, and the cells were covered with 0.9% methylcellulose in cell culture medium and cultured for two (Beta and Delta variants) or three days (WIV04 strain). The plaques were stained with 0.5% crystal violet for 10 min and then counted. The % neutralization = (1‐ sample plaque/negative control plaque) × 100%, and the PRNT_50_ values were determined using nonlinear regression analysis.

### Microneutralization Assay

Vero E6 cells (8 × 10^4^ per well) were seeded into 48‐well plates and cultured overnight. COVID‐HIG‐001, COVID‐HIG‐002, and COVID‐HIG‐003 were serially diluted by fivefold (maximum concentration, 5 mg mL^−1^) in minimum Eagle's medium for seven dilutions. Next, COVID‐HIG‐001, COVID‐HIG‐002, and COVID‐HIG‐003 were incubated with SARS‐CoV‐2 WIV04, Beta, or Delta strain (4000 TCID_50_ mL^−1^; 0.05 multiplicity of infection) for 1 h. Maltose (0.1%) was used as a negative control. Next, 100 µL of the virus and immunoglobulin mixture was incubated with the cells for 1 h to allow virus attachment. After extensive washing with phosphate‐buffered saline, the mixture was replaced with normal cell culture medium, and the cells were cultured for another 24 h. The collected cell supernatant was treated with lysis buffer (Takara, Shiga, Japan, Cat no. 9766) to detect virus copies. The % neutralization = (1‐ sample copies/negative control copies) × 100%, and IC_50_ values were determined using nonlinear regression analysis.

### Mouse Experiments

*Ethics Statement*: The in vivo efficacy experiments were approved by the Institutional Animal Care and Use Committee of the Wuhan Institute of Virology, Chinese Academy of Sciences (ethics number: WIVA01202001) and conducted within the Animal Biosafety Level 3 facility in the National Biosafety Laboratory (Wuhan), Chinese Academy of Sciences.

Adv5‐hACE2 (4 × 10^8^ TCID_50_) was intranasally inoculated into 12–14‐week‐old IFNAR^−/−^ C57BL/6 female mice after sufficient anesthesia. At 5 d after Adv5‐hACE2 infection, the mice were infected with SARS‐CoV‐2 WIV04 (1 × 10^6^ TCID_50_ mL^−1^) intranasally. The clinical symptoms and body weight of the mice were observed daily. At 6 d after infection, the animals were sacrificed, and the tissues were collected for further virus copy and pathological evaluation.

*RNA Extraction and real‐time reverse transcription‐PCR (qRT‐PCR)*: RNA in the cell supernatant was extracted with a TaKaRa MiniBEST Viral RNA/DNA Extraction Kit Ver.5.0. Total RNA from the tissues was converted to cDNA as previously described.^[^ ^28^ ^]^ For quantification, 2 µL of cDNA was used as a template for quantitative polymerase chain reaction (qPCR; Takara, catalog no. RR820A). The primers used for qPCR were: 5′‐CAATGGTTTAACAGGCACAGG‐3′ and 5′‐CTCAAGTGTCTGTGGATCACG‐3′. The qPCR experiment was performed as described previously.^[^ ^49^ ^]^

*Histochemical Staining*: The collected lung tissues were fixed in 10% neutral formalin buffer, embedded in paraffin, and sectioned (3 µm). The sections were stained with hematoxylin and eosin or subjected to immunohistochemistry assay. The lung pathology score was determined based on the pathological changes in the alveoli, bronchi, blood vessels, and other parts of the lungs. The scores were divided into six grades (Table S8, Supporting Information).

*Immunofluorescence Assay*: Tissue sections from COVID‐HIG‐treated and untreated mice lungs were blocked with 5% bovine serum albumin at room temperature for 2 h after dewaxing. The sections were incubated with primary antibody (rabbit anti‐SARS‐CoV‐2 nucleocapsid proteins polyclonal antibody, 1:500 dilution) for 2 h, followed by incubation with secondary antibody (Alexa 555‐labeled goat anti‐rabbit, 1:500 dilution). The nuclei were stained with Hoechst 33258 dye (Beyotime, Shanghai, China). Images were obtained using a JMX Panorama Scanner (Thermo Fisher Scientific, Waltham, MA, USA).

### Statistical Analysis

GraphPad Prism 9.0 (GraphPad, Inc., La Jolla, CA, USA) was used for statistical analysis. Unless otherwise indicated, the data are represented as the mean ± standard error (mean ± SEM) from three technical replicates. Sample size (*n*) for each statistical analysis was shown in figure legends. Body weight was analyzed using two‐way analysis of variance (ANOVA). Viral copy numbers were analyzed using unpaired t‐test or one‐way ANOVA. The dose‐inhibition curves and PRNT_50_ and IC_50_ values were determined by nonlinear regression using a dose‐response‐inhibition model with a variable slope. *P*‐values are represented in the figures as follows: *P < 0.05, **P < 0.01, ***P < 0.001.

## Conflict of Interest

X.M.Y., H.C.Y., and R.J. are employees of China National Biotec Group Company Limited; D.Y., H.L., J.Z.W., and Y.L.D. are employees of Chengdu Rongsheng Pharmaceuticals Co., Ltd; D.Y., H.L., T.J.L., Y.Z., C.Y.L., D.M.D., D.X.F., D.B.Z., Y.L.H., T.D., Y.X., and R.Z. are employees of Beijing Tiantan Biological Products Co., Ltd; C.S.L., Y.H., Y.P., R.H., Y.T.X., L.F., X.L.L., Z.J.Z., D.M. J., F.F.W., J.H.Y., K.P., D.M.X., and Y.L.H. are employees of Sinopharm Wuhan Plasma‐derived Biotherapies Co., Ltd. Sinopharm Wuhan Plasma‐derived Biotherapies Co. Ltd. filed patents on the production method of COVID‐HIG to China National Intellectual Property Administration. All other authors declare no conflict of interest. Figure 6b,c has been amended on May 16, 2022, after initial online publication.

## Author Contributions

D.Y., Y.F.L., H.L., and J.Z.W. contributed equally to this work. X.M.Y., H.C.Y., D.X.F., Y.L.H., D.B.Z., and C.S.L. initiated and coordinated the project. D.Y., H.L., M.L.W., J.Z.W., Y.H., Y.Z., and R.J. conceived and designed the experiments. D.M.X., R.H., Y.T.X., X.L.L., J.H.Y., Y.L.D., T.D., D.M.D., Y.X., and R.Z. collected plasma and prepared COVID‐HIG. H.L., J.Z.W., Y.H., and L.F. performed the FACS assay. Y.F.L conducted the BLI measurement of affinity and competition‐binding study. Z.J.Z., L.F., and K.D. performed the ELISA assays. Y.P. and D.M.J. conducted the pseudotyped virus neutralization activity experiment with the help of J.F.H., W.J.H., and L.D.G. Y.F.L., J.L., H.R.H., and Y.J.L. conducted animal experiments. J.L. and Y.F.L. conducted histopathology and immunohistochemistry assays. H.R.H. and Y.J.L. evaluated the authentic virus neutralizing activity. C.W.K. provided the SARS‐CoV‐2 Beta variant (NPRC 2.062100001). X.M.Y., M.L.W., C.S.L., Y.F.L., D.Y., H.L., Z.J.W., Y.H., T.J.L., F.F.W., and C.Y.L. completed the data analysis. Z.J.W., Y.F.L., D.Y., H.L., Y.H., Y.P., L.F., and T.J.L. wrote the paper. X.M.Y., M.L.W., and C.S.L. approved the final paper. All authors reviewed and edited the paper.

## Supporting information

Supporting Information

## Data Availability

The data that support the findings of this study are available from the corresponding author upon reasonable request.
