# Supplementary material for: Potent Anti‐SARS‐CoV‐2 Efficacy of COVID‐19 Hyperimmune Globulin from Vaccine‐Immunized Plasma
Source: Adv Sci (Weinh). 2022 Apr 11;9(14):2104333. doi: 10.1002/advs.202104333 (PMC9108634; doi:10.1002/advs.202104333)
Supplement: Supplementary file 1 — Supporting Information [file ADVS-9-2104333-s001.pdf]

Supporting Information

Potent Anti-SARS-CoV-2 Efficacy of COVID-19 Hyperimmune Globulin from Vaccine-Immunized Plasma

Ding Yu,[1,2†] Yu-Feng Li,[3†] Hong Liang,[1,2†] Jun-Zheng Wu,[1†] Yong Hu,[4] Yan Peng,[4] Tao-Jing Li,[2] Ji-Feng Hou,[5] Wei-Jin Huang,[5] Li-Dong Guan,[5] Ren Han,[4] Yan-Tao Xing,[4] Yong Zhang,[2] Jia Liu,[3] Lu-Feng,[4] Chun-Yan Li,[2] Xiao-Long Liang,[4] Ya-Ling Ding,[1] Zhi-Jun Zhou,[4] De-Ming Ji,[4] Fei-Fei Wang,[4] Jian-Hong Yu,[4] Kun Deng,[4] Dong-Mei Xia,[4] De-Mei Dong,[2] Heng-Rui Hu,[3] Ya-Jie Liu,[3] Dao-Xing Fu,[2] Yan-Lin He,[2,4] Dong-Bo Zhou,[2] Hui-Chuan Yang,[6] Rui Jia,[6] Chang-Wen Ke,[7] Tao Du,[2] Yong Xie,[2] Rong Zhou,[2] Ce-Sheng Li,[4]* Man-Li Wang,[3]* Xiao-Ming Yang[6]*

[1]Chengdu Rongsheng Pharmaceuticals Co., Ltd; Chengdu, China.
[2]Beijing Tiantan Biological Products Co., Ltd; Beijing, China.
[3]Center for Biosafety Mega-Science, Wuhan Institute of Virology, Chinese Academy of Sciences; Wuhan, China.
[4]Sinopharm Wuhan Plasma-derived Biotherapies Co., Ltd; Wuhan, China.
[5]National Institute for Food and Drug Control of China; Beijing, China.
[6]China National Biotec Group Company Limited; Beijing, China.
[7]Guangdong Provincial Center for Disease Control and Prevention; Guangzhou, China.

*Corresponding authors: Email: yangxiaoming@sinopharm.com (X.M.Y.); wangml@wh.iov.cn (M.L.W.); licesheng@sinopharm.com (C.S.L.).
†These authors contributed equally to this work.

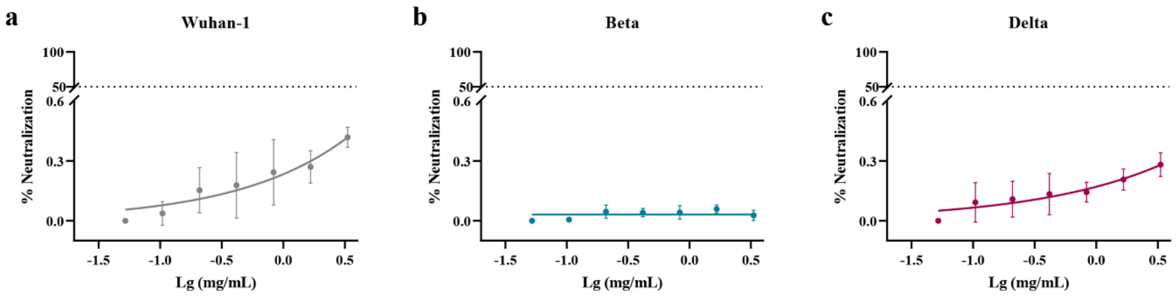

Figure S1. Assessment of the antiviral activity of IVIG against SARS-CoV-2 pseudotyped viruses *in vitro*. (a-c) IVIG showed no neutralization effect against pseudotyped SARS-CoV-2 Wuhan-1 (a), Beta (b), and Delta (c) strains. Pseudotyped viruses were pre-incubated with serial dilutions of COVID-HIG at different concentrations for 1 h at 37°C. Next, Huh-7 cells were incubated with the pseudotyped viruses for 24 h. Luciferase was detected to assess infection. The y-axis represents percent inhibition. Data are shown as mean ± SD of three independent experiments (n = 3). IVIG, human immune globulin intravenous.

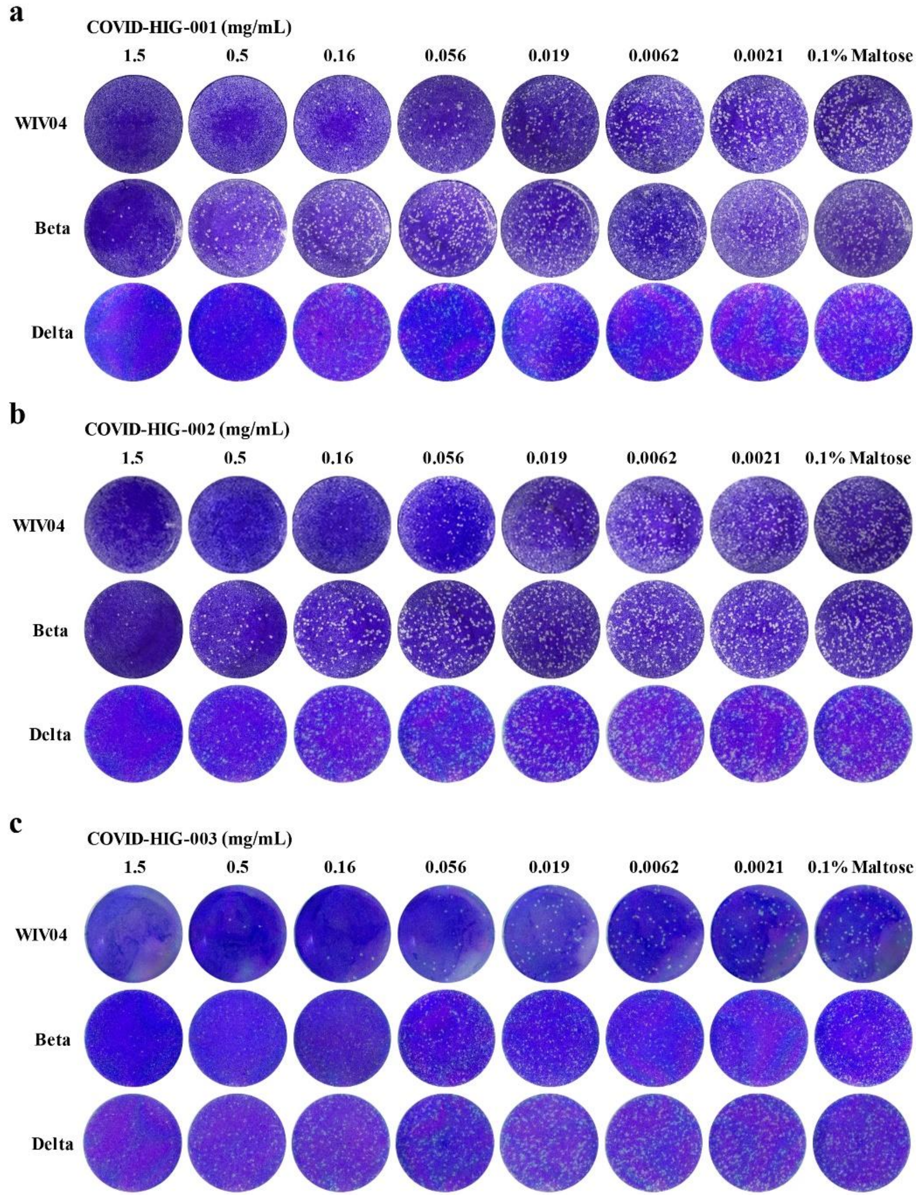

Figure S2. Plaque reduction neutralization assay of COVID-HIG. COVID-HIG-001 (a), COVID-HIG-002 (b), and COVID-HIG-003 (c) significantly inhibited infection by SARS-CoV-2 WIV04, Beta, and Delta strains in Vero E6 cells. Maltose was used as a negative control. Viruses were incubated with COVID-HIG at different concentrations at 37℃ for 1 h. Thereafter, Vero E6 cells were infected with SARS-CoV-2 WIV04, Beta and Delta strains. The infected cells were stained with hematoxylin/eosin at 48 h (Beta and Delta strains) or 72 h (WIV04 strain) post-infection. WIV04, nCoV-2019BetaCoV/Wuhan/WIV04/2019.

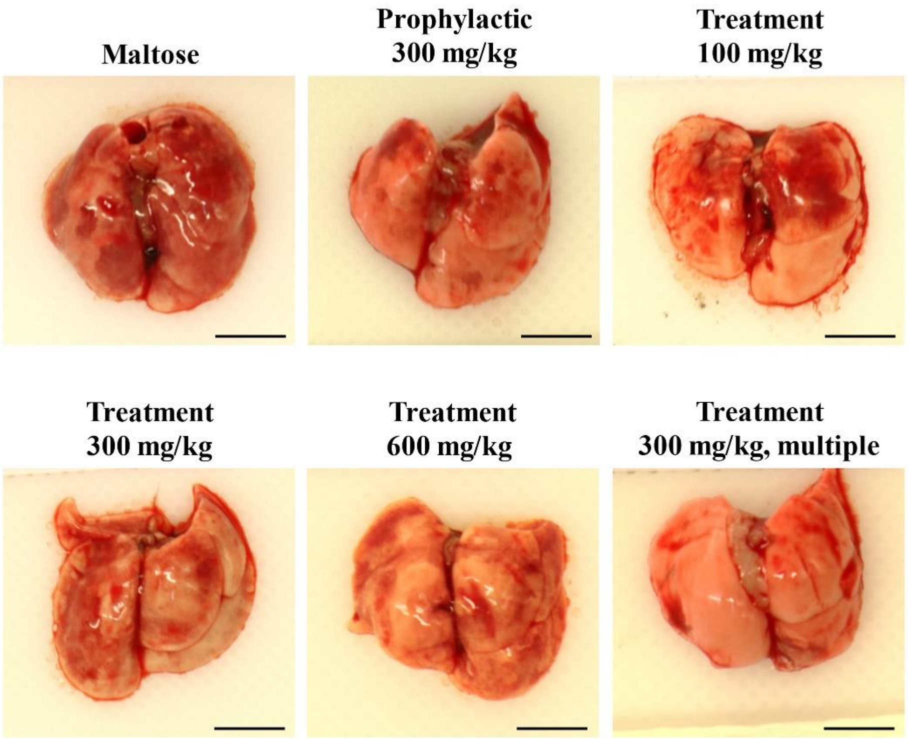

Figure S3. Gross observation of the lungs of mice treated with COVID-HID 6 days after SARS-COV-2 infection. Mice in the adjuvant control group had a large range of diffuse crimson lesions in the lungs, whereas the area of crimson lesions in the lungs of mice in the COVID-HIG prevention and treatment groups was significantly reduced. Maltose adjuvant-treated mice were used as the control group. Scale bar = 0.5 cm.

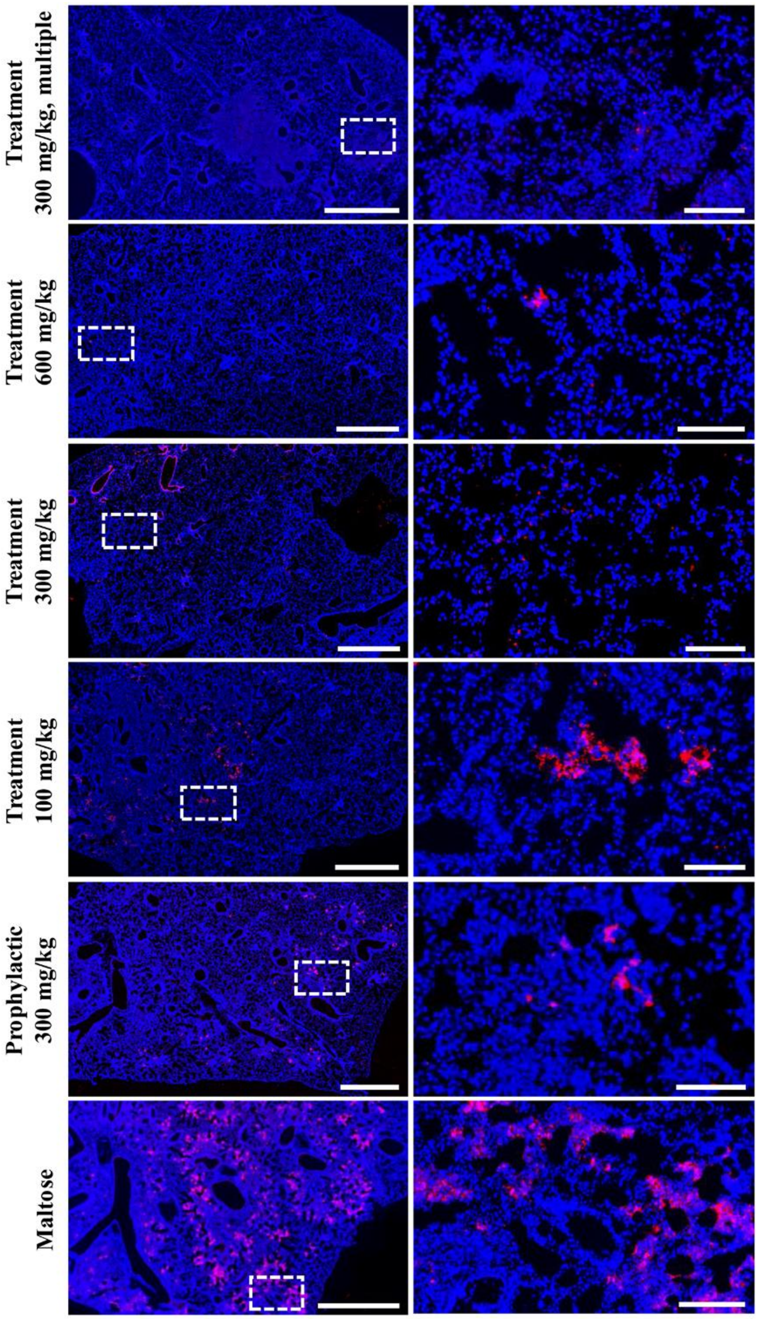

Figure S4. Virological analyses of treated and untreated mouse lungs using immunofluorescence staining of SARS-CoV-2 nucleocapsid proteins. Changes in virus protein expression (red fluorescence) in the mouse lungs. Tissues were dissected and collected for fixation, embedding, slicing, and immunofluorescence detection. Maltose-treated mice were used as the adjuvant control group. The figure on the right (scale bar = 100 nm) is an enlarged view of the white dotted box in the figure on the left (scale bar = 1 μm).

Table S1. Total protein and IgG concentrations of PBP and COVID-HIG

Lot	Product	Total protein (mg/mL)	IgG (mg/mL)
**COVID-HIG-001**	PBP	64.7	10.0
	COVID-HIG	53.2	52.1
**COVID-HIG-002**	PBP	63.0	9.8
	COVID-HIG	53.0	52.0
**COVID-HIG-003**	PBP	64.9	8.8
	COVID-HIG	54.2	53.5

IgG, immunoglobulin G. PBP, pooled BBIBP-CorV plasma.

Table S2. IgG subclass distribution of COVID-HIG

**COVID-HIG Lot**	**Distribution of the four IgG subclasses (%) ***			
	IgG1	IgG2	IgG3	IgG4
**COVID-HIG-001**	67.38%	28.09%	2.61%	1.93%
**COVID-HIG-002**	64.91%	29.64%	3.32%	2.13%
**COVID-HIG-003**	65.27%	29.05%	3.51%	2.17%
**Mean ± SD**	65.85% ± 1.33%	28.93% ± 0.78%	3.14% ± 0.48%	2.08% ± 0.13%

*Immunoturbidimetry was used for detection.

Table S3. Summary values of binding affinity, blocking potency, and neutralization potency of COVID-HIG-003

Strain	K_D (nM)		IC_{50} (nM) of ELISA	IC_{50} (nM) of PBNA	$PRNT_{50}$ (nM)	Ratio ($K_D/PRNT_{50}$)
WIV04 (Or Wuhan-1)	S protein	4.60	2.27×10^3	0.55×10^3 (Wuhan-1)	0.19×10^3	2.4×10^{-2}
	NTD	14.80				7.8×10^{-2}
	NP	14.30				N/A
	RBD	99.50				5.2×10^{-1}
Beta	RBD	5.33	4.66×10^3	3.95×10^3	1.44×10^3	3.7×10^{-3}
Delta	RBD	21.90	1.13×10^3	1.52×10^3	1.61×10^3	1.4×10^{-2}

WIV04, nCoV-2019BetaCoV/Wuhan/WIV04/2019 strain. S, spike. NTD, N-terminal domain of S protein. NP, nucleocapsid protein. RBD, receptor-binding domain of S protein. K_D, equilibrium dissociation constant. Competitive ELISA assays were performed to calculate the 50% inhibitory concentration values for the blocking ability of COVID-HIG. PBNA, pseudotyped virus-based neutralization assay. $PRNT_{50}$, the 50% plaque reduction neutralization test concentration of COVID-HIG against strains. N/A, not applicable.

Table S4. Neutralization potency of PBP and COVID-HIG against the pseudotyped SARS-CoV-2 Wuhan-1 strain

Lot	Product	IC_{50} (mg/mL)
**COVID-HIG-001**	PBP	0.2759
	COVID-HIG	0.0856
**COVID-HIG-002**	PBP	0.2537
	COVID-HIG	0.0693
**COVID-HIG-003**	PBP	0.4582
	COVID-HIG	0.0827

Wuhan-1, SARS-CoV-2 Wuhan-Hu-1 strain. PBP, pooled BBIBP-CorV plasma.

Table S5. Gross pathological findings

Group	ID #	Severity grade*				Individual#	Group mean
		Alveolar wall	Alveolar cavity	Bronchus	Blood vessel		
Maltose	1	+++	++	+++	+++	5	5.0
	2	+++	+++	+++	+++	6	
	3	+++	+++	+++	+++	6	
	4	+++	++	+++	+++	5	
	5	+++	+++	+++	+++	6	
	6	+++	++	+++	+++	5	
	7	++	++	++	+++	4	
	8	+	+	++	++	3	
COVID-HIG 300 mg/kg, -24h	1	+	-	++	++	2	2.8
	2	++	++	+	-	2	
	3	++	++	++	++	3	
	4	++	++	++	+++	4	
	5	-	-	-	+	1	
	6	+	+	++	+++	3	
	7	++	++	+++	++	4	
	8	+	++	++	+++	3	
COVID-HIG 300 mg/kg, multiple	1	+	+	+++	+++	4	2.9
	2	+	-	+	++	2	
	3	++	+	++	+++	3	
	4	+	++	++	+++	4	
	5	++	+++	+++	+++	5	
	6	+	-	+	+	1	
	7	++	++	++	++	3	
	8	-	-	-	+	1	
COVID-HIG 600 mg/kg, +2 h	1	+	+	++	+++	3	3.2
	2	+	++	+++	++	4	
	3	+	++	++	+++	4	
	4	+++	++	++	++	4	
	5	+	-	+	+	1	
	6	+	+	+	++	2	
	7	+	+	+++	++	3	
	8	++	+++	+++	+++	5	
COVID-HIG 300 mg/kg, +2 h	1	++	+	++	+++	4	3.9
	2	+++	+++	+++	+++	6	
	3	+	-	+	+	1	
	4	++	++	++	+++	4	
	5	++	++	++	+++	4	
	6	++	++	++	+++	4	
	7	++	+++	+++	++	4	
	8	++	+	++	+++	4	
COVID-HIG 100 mg/kg, +2 h	1	+++	++	+++	+++	5	3.6
	2	+++	+++	++	+++	5	
	3	+	+	++	+++	3	
	4	++	++	++	+++	4	
	5	+++	++	++	+++	5	
	6	++	++	+	+++	4	
	7	+	-	-	+	1	
	8	+	+	+	++	2	

*In a single score, - indicates no pathological changes; + indicates mild lesions; ++ indicates moderate lesions; +++ indicates severe lesions. #The comprehensive score was assigned

according to the standards introduced in the Materials and Methods; 0, no lesion; 1–2, mild lesions; 3–4, moderate lesions; 5–6, severe lesions.

-24 h, COVID-HIG prophylactic groups; +2 h, COVID-HIG treatment groups.

Table S6. RBD-IgG titer of PBP and COVID-HIG

Lot	Product	RBD-IgG Titer
**COVID-HIG-001**	PBP	1:944
	COVID-HIG	1:5,184
**COVID-HIG-002**	PBP	1:674
	COVID-HIG	1:5,919
**COVID-HIG-003**	PBP	1:551
	COVID-HIG	1:4,737

RBD, receptor-binding domain; PBP, pooled BBIBP-CorV plasma.

Table S7. Basic information of pseudotyped SARS-CoV-2 viruses

**Pseudotyped virus**	***S* gene source**	**Spike mutation site**	**Manufacturer**
Wuhan-1	GenBank: MN908947	/	Gobond Science and Technology (Beijing) Co., Ltd.
D614G		D614G	
Alpha		delta69-70HV, delta144Y, N501Y, A570D, D614G, P681H, T716I, S982A, D118H	
Beta		L18F, D80A, D215G, delta242-244LAL, R246I, K417N, E484K, N501Y, D614G, A701V	
Gamma		L18F, T20N, P26S, D138Y, R190S, K417T, E484K, N501Y, D614G, H655Y, T1027I, V1176F	
Kappa		G142D, E154K, L452R, E484Q, D614G, P681R, Q1071H, H1101D	
Delta		T19R, G142D, F157del, R158del, L452R, T478K, D614G, P681R, D950N	
Omicron		A67V, H69del, V70del, T95I, G142D, V143del, Y144del, Y145del, N211del, L212I, ins214EPE, G339D, S371L, S373P, S375F, K417N, N440K, G446S, S477N, T478K, E484A, Q493R, G496S, Q498R, N501Y, Y505H, T547K, D614G, H655Y, N679K, P681H, N764K, D796Y, N856K, Q954H, N969K, L981F	

Wuhan-1, SARS-CoV-2 Wuhan-Hu-1 strain.

Table S8. Gross severity grades recorded for the lung based on the percent lobe affected by gross changes

Pathological change score	Judgment basis	Evaluation of damage rating
6	More than 75% of the lungs had diffuse alveolar injury; the alveolar wall was significantly thickened, an alveolar hyaline membrane was formed, and fibrosis was significant; most small bronchial epithelium proliferated and dropped off considerably; edema and hyperplasia of vascular endothelial cells were significant; inflammatory cell infiltration was diffuse.	Severe
5	Here, 51%–75% of the lung had diffuse alveolar injury; alveolar wall thickening was obvious, an alveolar hyaline membrane was formed, and fibrosis was obvious; most small bronchial epithelium proliferated and dropped off considerably; edema and hyperplasia of vascular endothelial cells were obvious; inflammatory cell infiltration was diffuse	
4	Here, 36%–50% of the lung had moderate injury; some alveolar walls were thickened, alveolar fibrin exudation and fibrosis were obvious; some bronchiolar epithelium hyperplasia and necrosis were present; edema and hyperplasia of some vascular endothelial cells; significant infiltration of inflammatory cells.	Moderate
3	In this case, 21%–35% of the lung had mild to moderate injury; some alveolar walls were thickened; fibrin exudation and fibrosis were rare in alveoli; partial bronchiolar epithelial hyperplasia was observed; significant infiltration of inflammatory cells was present	
2	Here, 5%–20% of the lung had mild pathological injury; most alveolar walls and alveolar cavities had normal structures; a small part of the alveolar walls was thickened; fibrin exudation and fibrosis were seen in alveoli; small amount of bronchiolar epithelium hyperplasia was observed; a small amount of monocyte and lymphocyte infiltration was observed.	Mild
1	Finally, <5% of the lung had slight pathological injury; most alveoli, bronchi, and blood vessels had normal structures; very few alveolar walls were thickened; fibrin exudation and fibrosis were rare in alveoli; individual bronchiolar epithelial hyperplasia was observed; a small amount of mononuclear and lymphocyte infiltration was occasionally was observed.	
0	The alveolar structure was intact; there was no inflammatory infiltration and no congestion; the bronchial and vascular structures were normal.	None
